# Single nucleotide polymorphisms and sporadic colorectal cancer susceptibility: a field synopsis and meta-analysis

**DOI:** 10.1186/s12935-018-0656-2

**Published:** 2018-10-10

**Authors:** Jing Wen, Qian Xu, Yuan Yuan

**Affiliations:** 1 Tumor Etiology and Screening Department of Cancer Institute and General Surgery, The First Hospital of China Medical University, No.155 NanjingBei Street, Heping District, Shenyang, 110001 Liaoning China; 2 Key Laboratory of Cancer Etiology and Prevention in Liaoning Education Department, The First Hospital of China Medical University, Shenyang, 110001 China; 3 Key Laboratory of GI Cancer Etiology and Prevention in Liaoning Province, The First Hospital of China Medical University, Shenyang, 110001 China

**Keywords:** Non-hereditary colorectal cancer, Single nucleotide polymorphisms, Field synopsis, Meta-analysis

## Abstract

**Background:**

Although mounting non-hereditary colorectal cancer (NHCRC) associated single nucleotide polymorphisms (SNPs) have been observed, no field synopsis and meta-analysis has been conducted through systematically assessing cumulative evidence, during the past 5 years.

**Methods:**

We retrieved the database via the PubMed, Web of Science and Embase gateways to identify publications concerning the associations between SNPs and risk of NHCRC, up to May 1st, 2017. To assess the finding credibility, cumulative evidence was graded based on the Venice criteria. Meta-analysis was also performed for three subgroups including ethnicity (Asian vs Caucasian), primary cancer site (colon vs rectum) and TNM stage (I II vs III IV). Then, we arranged those high quality SNPs into different regions according to their locations on genes to evaluate their functional roles on CRC development.

**Results:**

5114 publications were collected and 1001 of them met our inclusion criteria, which totally included 1788 SNPs in 793 genes or distinct chromosomal loci. Totally, we performed 359 primary and subgroup meta-analyses for 160 SNPs in 96 distinct genes. By utilizing the Venice criteria, we identified 15 high quality SNPs with 25 high credibility significant associations. Furthermore, we artificially divided the high quality SNPs into different groups, based on their SNP loci (exon region, intron region, promoter region, downstream region, non-coding region and intergenic region).

**Conclusion:**

We have identified 15 high quality SNPs which may act as promising genetic biomarkers for clinical NHCRC susceptibility screening and explored their functional roles on the NHCRC development based on their locations on genes.

**Electronic supplementary material:**

The online version of this article (10.1186/s12935-018-0656-2) contains supplementary material, which is available to authorized users.

## Background

Colorectal cancer (CRC) is the third most frequent cancer and the fourth major cause of cancer death worldwide [1]. Genetic factors play an important role in the carcinogenesis of CRC. Traditionally, CRC can be divided into familial CRC (hereditary CRC, HCRC) and sporadic CRC (non-hereditary CRC, NHCRC). HCRC only accounts for 20–25% of all CRC and is mainly attributed to precise high-penetrance mutations [2]. The overwhelming majority of CRC is NHCRC that can be caused by some genetic defects like single nucleotide polymorphism rather than any exact genetic mutation. Understanding of genetic variation is beneficial to strengthen the precaution, screening and early diagnosis of CRC, which is not only for HCRC but also for NHCRC. In a sense, the prediction and control of NHCRC is more expected than HCRC because it occupies the majority of CRC, and the control measures may be feasible and operable.

Single nucleotide polymorphism (SNP) is a common genetic variation, which may result in different functional products, thus affecting individual susceptibility to diseases. Hence, SNP can be considered as biomarker to predict the risk of sporadic tumor including CRC. During the past three decades, numerous SNPs have been illustrated to be correlated with CRC risk by extensive genome-wide association studies (GWAS) and also candidate-gene association studies (CGAS). Different data based Meta analyses from different angles also reported in the genetic predisposition to NHCRC. Making a general observation of preceding meta-analyses, most of them gathered only a fraction of SNPs and few noticed complete picture of SNPs in NHCRC from a field perspective. It’s worth noting that, there have existed two comprehensive field meta-analyses which demonstrated all CRC risk associated variants, up to 2012, providing directions for future investigators [3, 4]. Inspired by these two articles, we noticed that SNP plays an essential role in the genetic predisposition of CRC, constituting nearly 80% of he significant genetic variants which also include the insertion/deletion polymorphism and variable number of tandem repeat (VNTR). For SNP only, a renewed field synopsis and meta-analysis is required on account of the past 5 years since the latest field synopsis published, and the heterogeneity from ethnicity, primary cancer site and TNM stage must be considered. What’s more, no studies mentioned the role of the whole associated SNPs on CRC development, based on their locations on genes.

In the present systematic review and meta-analysis, we focus on the high quality SNPs (which mean the SNPs are statistically associated with CRC risk in high credibility level, assessed by Venice criteria) in the field of genetic predisposition to NHCRC, involving the correlations of SNPs with ethnicity, primary cancer site (colon or rectal) and TNM stage (I II or III IV). Then, we arranged those high quality SNPs into different regions according to their locations on genes to evaluate their functional roles on CRC development.

## Materials and methods

### Retrieval strategy

A comprehensive systematic literature search was performed for the publications concerning the association between SNP and risk of NHCRC. We retrieved the database via the PubMed, Web of Science and Embase gateway by using the search terms “(polymorphism or “single nucleotide polymorphism” or SNP or “genome wide association study” or GWAS) and (colon or rectal or rectum or colorectal) and (cancer or tumor or carcinoma or neoplasm)”, up to May 1st, 2017. Moreover, each identified SNP was adopted as a keyword to further improve the search, for instance, ‘XPG’ or ‘rs17655’ in combination with “(colon or rectal or rectum or colorectal) and (cancer or tumor or carcinoma or neoplasm)” as query term.

### Inclusion and exclusion criteria

**Fig. 1:**
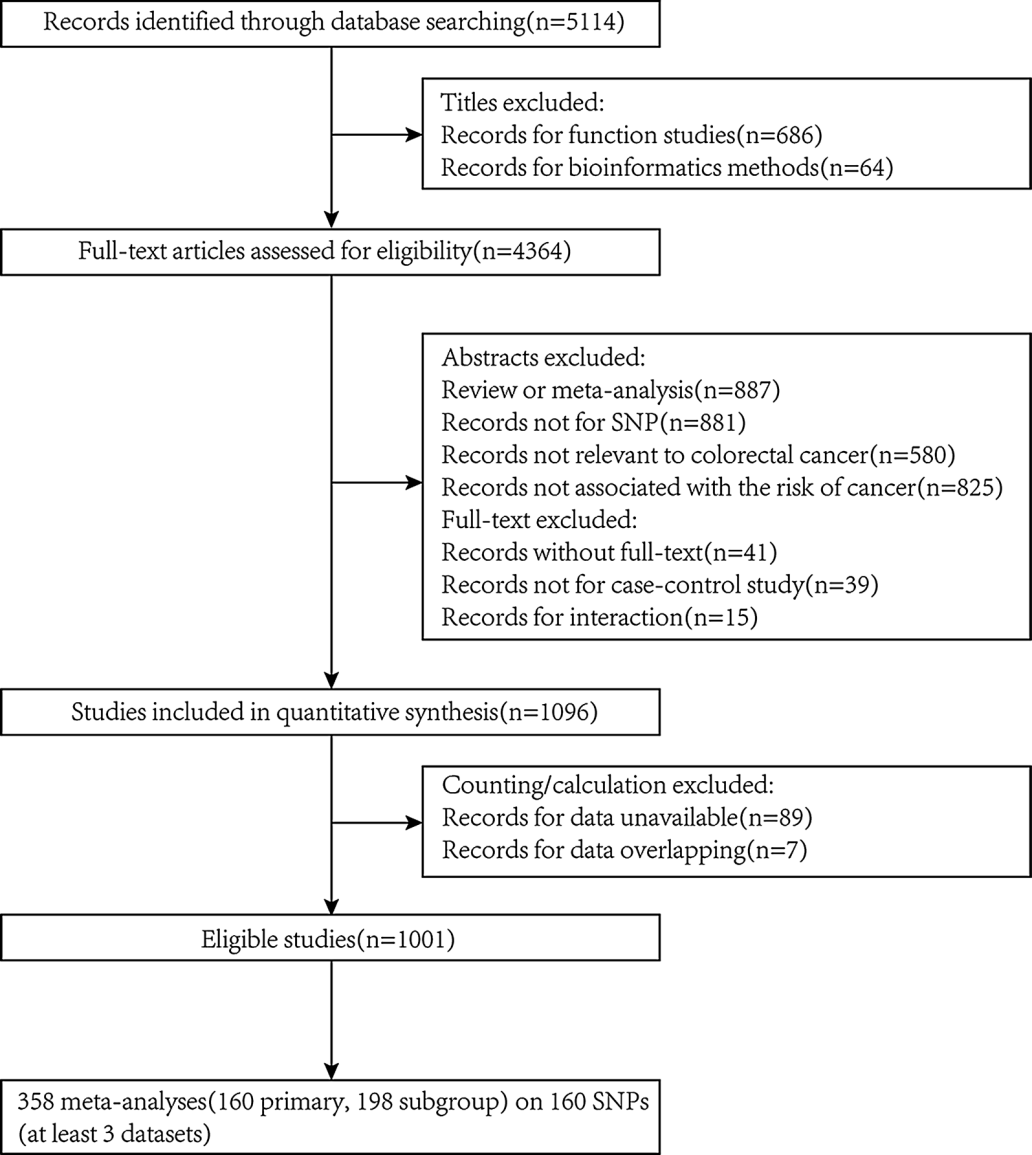
Flow diagram for selecting eligible studies

To identify all eligible studies, we adopted the following inclusion criteria: (1) case–control study either candidate-gene association studies (CGAS) or genome-wide association studies (GWAS); (2) explored the correlation between SNP and NHCRC. In addition, the main exclusion criteria were: (1) overlapping studies; (2) no relation to NHCRC or nothing concerning SNPs; (3) no available data or inadaptable SNP genotyping methods; (4) any research published in abstraction form solely (e.g. conference proceedings or scientific meetings) (Fig. 1).

### Data extraction

Data were independently extracted by two of the authors (Jing Wen and Qian Xu). Items collected from all eligible publications included first author, publication year (unpublished data show study year), race of participants, sample size, genes, SNP locus, genotype counts of cases and controls and HWE in controls. Multiple populations comprising one publication were extracted individually. Concerning GWAS, discovery and replication studies were regarded as separate datasets and were also extracted individually. When it came to eligible articles along with unreported data, we made efforts to contact with the authors.

### Assessment of cumulative evidence

The epidemiology credibility of all seemingly significant associations confirmed by our meta-analysis were taken into account by applying Venice criteria [5, 6]. Three categories considered as fundamental criteria to defined the credibility level are as follows:

1. Amount of evidence was evaluated by the total number of both cases and controls expressing the test alleles or genotypes: category ‘A’, ‘B’, ‘C’ represent for large-scale, moderate, or little respectively with over 1000, 100–1000 and less than 100 sample size.
2. Replication was classed based on the statistic of heterogeneity: category ‘A’, ‘B’, ‘C’ respectively stand for little inconsistency, moderate inconsistency or large inconsistency (no association) with I^2^ < 25%, 25–50% and > 50%.
3. Protection from bias was classed as ‘A’ with no bias which was improbably to explain the positive result of association, ‘B’ with no obvious bias but could be the reason for the association, or ‘C’ with demonstrable bias. The general checks for bias include: association lost with removal of initial study; small intensity of association (0.87 < OR < 1.15) and existence of publication bias [7, 8].

According to criteria mentioned above, the accumulative evidence of associations calculated by meta-analysis were regarded as high credibility level (three grades ‘A’), intermediate credibility level (either ‘A’ or ‘B’), and low credibility level (if any grades ‘C’). Notably, the heterogeneity and bias could be exempted if the *P* value < 1×10^−7^ after removing the initial study [8].

### Statistics

Statistical analyses in our study were conducted by STATA software, version 11.0 (STATA Corp., College Station, TX, USA). All tests were two-tailed and *P* values ≤ 0.05 were regarded as the statistical significance level only if we emphasized once more. And it would reach a genome-wide significance level if *P *< 5 × 10^−8^ [9]. The Hardy–Weinberg equilibrium (HWE) among genotype distributions of controls was assessed by Chi square test and *P* values < 0.05 were regarded as statistically significant disequilibrium. Appraisals of the association between the SNPs and colorectal cancer risk were assessed by pooled odds ratios (ORs) and 95% confidence intervals (CIs) calculated by random effect models when heterogeneity of between-study exists [10], otherwise fixed effect model [11]. Begg’s test, as a funnel plot analyses, was implemented to verify significant asymmetry [12] and the modified Egger’s test owns the capacity to correct type I errors through evaluating bias caused by small studies [13]. *P* value less than 0.10 was regarded as the threshold in both Begg’s or Egger’s test.

In addition, *q* value was considered as a measure for statistically significant findings in terms of false discovery rate (FDR), which is the proportion that significant findings are truly null hypotheses. For instance, 5% false discovery rate means that among all statistically significant SNPs, 5% of them are not actually associated with CRC risk. And we also considered 0.05 as the threshold of q value [14, 15].

## Results

### Features of eligible studies

According to the screening process showed in Fig. 1, 5114 publications were collected and 1001 of them met our inclusion criteria, which totally included 1788 SNPs in 793 genes or distinct chromosomal loci with 2,200,290 subjects extracted (cases: 971,074, ratio: 44%, range: 8–10,409, mean: 550).

Based on the ethnicity of study population, investigations for Caucasian (57%) were slightly more than those for Asian. Besides, over a quarter of the available articles detailed the primary site (colon vs rectum) of colorectal cancer, and the articles that mentioned TNM stage of UICC/AJCC also account for 13%. Additionally, nearly a half of the investigated SNPs were exonic SNPs (45%), others were located in intron (20%), 3′-UTR (4%), 5′-UTR (1%), upstream (14%), downstream regions (2%) non-coding (7%) or intergenic regions (6%).

### Meta analysis findings

Totally, we performed 359 meta-analyses for 160 SNPs in 96 distinct genes. Each meta-analysis involved at least three studies (CGAS or GWAS) with available co-dominant genotypes and HWE. Of these, 160 were primary meta-analyses and 199 were subgroups meta-analyses defined by ethnicity (Caucasian, n = 90; Asian, n = 53), primary cancer site (colon, n = 22; rectum, n = 14) and TNM stage (I II, n = 10; III IV, n = 10). Of the 359 meta-analyses conducted, 90 (25%) attained statistically significant findings, other 269 being non-significant. Furthermore, 40.3% (n = 145) had little or no heterogeneity (I^2^ < 25%), 14.2% (n = 51) had moderate heterogeneity (25% ≤ I^2^ ≤ 50%), and 45.5% (n = 164) had large heterogeneity (I^2^ > 50%). Comparing the proportion of large study heterogeneity, we found that it was significantly lower for 90 positive SNPs than the remaining SNPs (19.8% and 46.5%, respectively; *P* value = 6.88E−6). Evaluation of publication bias conducted for all meta-analyses showed that totally 52 of them had statistically significant publication bias, 15 for ethnicity subgroup, 4 for cancer sites subgroup and 2 for TNM stage subgroup. In sensitivity analyses, eight SNPs was no longer significant after removing one record from meta-analysis and five of them showed more than 5% alteration of OR value (rs3918242, rs1048943, rs5498, rs4444903, rs10808556).

Comprehensively considering the impact of the evidence amount, replication consistency (heterogeneity), and protection from bias (derived from publication bias, initial study influence and OR value) on the cumulative evidence, we applied the Venice criteria that could assess the epidemiological credibility for all significant findings. Thus, the high, intermediate and low credibility level of cumulative evidence were detected, which respectively account for 28% (n = 25), 16% (n = 14), 56% (n = 51). Publication bias was the most common cause (41/65) for non-high-quality evidence, and the inter-study heterogeneity could be the second (33/65). From the 25 high credibility significant associations, we identified 15 distinct high quality SNPs which were presented in Fig. 5.

#### Results from whole population analysis

Significant associations in primary meta-analysis are reported in Table 1, characterized by high (n = 10), intermediate (n = 3) or low quality (n = 23). Seven of the ten high quality SNPs reached a genome-wide significance level, *P *< 5 × 10^−8^ (BMP2 rs961253, CASC8 rs1505477, BMP4 rs4444235, SMAD7 rs12953717, CCAT2 rs6983267, TGF-β1 rs1800469, LOC105376400 rs10795668 and GREM1-SCG5 rs4779584). Other three high quality SNPs were: GREM1-SCG5 rs4779584, ADIPOQ rs2241766 and miR-27a rs895819.

**Table 1:** Genetic variants associated with NHCRC after meta-analyses of at least three independent datasets (primary meta-analyses)

Gene	SNPs	Chr	Alleles^a^	Datasets	Cases	Controls	OR	CI-U	CI-L	P	FDR	Venice criteria	Level of evidence
BMP2	rs961253	20	C-A	23	40507	40740	1.121	1.098	1.144	7.77E−27	7.15E−25	AAA	High
CASC8	rs10505477	8	T-C	13	12933	13259	0.856	0.828	0.886	9.27E−19	2.84E−17	AAA	High
BMP4	rs4444235	14	T-C	27	39030	39934	1.083	1.061	1.105	8.24E−15	1.89E−13	AAA	High
SMAD7	rs12953717	18	C-T	9	10782	10011	1.163	1.118	1.209	4.93E−14	8.00E−13	AAA	High
CCAT2	rs6983267	8	G-T	26	33098	30415	0.845	0.807	0.884	3.35E−13	3.86E−12	AAA	High
TGF-β1	rs1800469	19	C-T	21	17404	36234	0.911	0.887	0.936	1.59E−11	1.62E−10	AAA	High
LOC105376400	rs10795668	10	G-A	8	5744	5481	0.837	0.791	0.886	8.43E−10	5.27E−09	AAA	High
GREM1-SCG5	rs4779584	15	C-T	16	25151	24548	1.154	1.093	1.217	1.69E−07	8.62E−07	AAA	High
ADIPOQ	rs2241766	3	T-G	7	2400	2972	1.216	1.115	1.326	9.33E−06	3.07E−05	AAA	High
miR-27a	rs895819	19	T-C	5	1562	1852	1.192	1.076	1.320	0.001	0.002	AAA	High
ADIPOR1	rs1342387	1	G-A	5	1843	2563	0.824	0.755	0.900	1.82E−05	5.76E−05	ABA	Intermediate
ICAM1	rs5498 469	19	A-G	3	358	335	0.740	0.595	0.920	0.007	0.012	BAA	Intermediate
PARP-1	rs1136410	1	T-C	3	808	1849	1.181	1.043	1.337	0.009	0.015	ABA	Intermediate
RHPN2	rs10411210	19	C-T	15	22299	23280	0.881	0.844	0.919	6.12E−09	4.69E−08	ABC	Low
SMAD7	rs4464148	18	T-C	8	9736	8573	1.142	1.091	1.196	1.62E−08	1.15E−07	AAC	Low
SMAD7	rs4939827	18	T-C	17	16336	15443	0.859	0.813	0.908	5.94E−08	3.42E−07	ACA	Low
CDH1	rs9929218	16	G-A	17	22459	24079	0.930	0.903	0.958	1.51E−06	5.80E−06	AAC	Low
COLCA1	rs3802842	11	A-C	18	18043	17876	1.149	1.086	1.216	1.62E−06	5.97E−06	ACC	Low
NA	rs719725	9	A-C	14	11820	13119	0.934	0.900	0.968	2.17E−04	5.56E−04	AAC	Low
MLH1	rs63750447	3	T-A	6	1427	1491	2.449	1.426	4.205	0.001	0.002	CCC	Low
XPG	rs17655	13	G-C	8	4752	5648	1.101	1.036	1.170	0.002	0.004	AAC	Low
NAT2	rs1801280	8	T-C	3	2066	2581	0.876	0.807	0.952	0.002	0.004	AAC	Low
NA	rs11568820	12	G-A	5	4278	4693	1.112	1.036	1.194	0.003	0.006	AAC	Low
VDR	rs1544410	12	G-A	14	10404	11213	0.768	0.633	0.930	0.007	0.012	ACC	Low
CCND1	rs9344	11	G-A	21	4757	6680	1.119	1.026	1.220	0.011	0.017	ACC	Low
CDH1	rs16260	16	C-A	8	6062	7045	0.934	0.884	0.988	0.016	0.023	AAC	Low
EGF	rs4444903	4	G-A	8	1234	1377	0.819	0.692	0.969	0.020	0.027	ACC	Low
GREM1	rs16969681	15	C-T	6	7300	9039	1.157	1.022	1.311	0.021	0.028	ACA	Low
MTRR	rs1801394	5	A-G	19	8409	11893	1.046	1.004	1.089	0.030	0.036	ABC	Low
NQO1	rs1800566	16	C-T	13	6016	6905	1.142	1.013	1.288	0.030	0.036	ACC	Low
MMP9	rs3918242	20	C-T	5	829	1096	0.803	0.657	0.980	0.031	0.037	BAC	Low
ERCC1	rs11615	19	C-T	5	982	1251	1.147	1.011	1.302	0.033	0.037	AAC	Low
APC	rs459552	5	A-T	13	9440	10200	0.950	0.905	0.997	0.037	0.041	AAC	Low
CYP1A1	rs1048943	15	A-G	13	3509	3960	1.287	1.011	1.637	0.040	0.043	ACA	Low
GH1	rs2665802	17	T-A	3	2740	3198	0.929	0.863	0.999	0.046	0.047	AAC	Low
RETN	rs1862513	19	C-G	3	1013	1049	1.145	1.000	1.311	0.050	0.050	ABC	Low

*Chr* chromosome, *FDR* false discovery rate

^a^Major alleles-minor alleles; Venice criteria: A (high), B (moderate), C (weak) credibility for three parameters (amount of evidence, heterogeneity and bias; see text and Additional file 1 for more details); level of evidence: overall level of summary evidence according to the Venice criteria

#### Results from subgroup analyses

Significant associations in subgroup analyses were shown in Table 2, featured with high (n = 15), intermediate (n = 11) or low quality (n = 28). Results from three stratification analyses (ethnicity, primary cancer site and TNM stage) were illustrated as follows.

**Table 2:** Genetic variants associated with NHCRC after meta-analyses of at least three independent datasets (subgroup meta-analyses)

Gene	SNPs	Chr	Alleles^a^	Datasets	Subgroups	Cases	Controls	OR	CI-U	CI-L	P	FDR	Venice criteria	Level of evidence
TGF-β1	rs1800469	19	C-T	16	Asian	14494	32550	0.911	0.885	0.938	2.43E−10	2.03E−09	AAA	High
LOC105376400	rs10795668	10	G-A	4	Asian	2479	2659	0.803	0.741	0.872	1.37E−07	7.41E−07	AAA	High
KRAS	rs712	12	G-T	3	Asian	1355	1219	1.405	1.226	1.610	9.99E−07	4.37E−06	AAA	High
ADIPOR1	rs1342387	1	G-A	3	Asian	1218	1724	0.779	0.697	0.870	9.22E−06	3.07E−05	AAA	High
ADIPOQ	rs2241766	3	T-G	5	Asian	2143	2666	1.215	1.111	1.328	6.03E−05	1.63E−04	AAA	High
miR-196a2	rs11614913	12	C-T	4	Asian	1397	2040	0.836	0.757	0.922	3.54E−04	8.79E−04	AAA	High
BMP2	rs961253	20	C-A	21	Caucasian	38172	38356	1.115	1.092	1.139	3.57E−24	1.64E−22	AAA	High
BMP4	rs4444235	14	T-C	22	Caucasian	34181	34254	1.086	1.063	1.110	5.22E−14	8.00E−13	AAA	High
SMAD7	rs12953717	18	C-T	8	Caucasian	10640	9845	1.161	1.116	1.208	1.16E−13	1.53E−12	AAA	High
GREM1-SCG5	rs4779584	15	C-T	12	Caucasian	21029	19867	1.16	1.092	1.232	1.45E−06	5.80E−06	AAA	High
LOC105376400	rs10795668	10	G-A	4	Caucasian	3265	2822	0.87	0.804	0.942	0.001	0.002	AAA	High
CCND1	rs9344	11	G-A	7	Rectum	775	2241	1.272	1.126	1.436	1.05E−04	2.77E−04	AAA	High
MTHFR	rs1801131	1	A-C	6	Rectum	1625	2987	0.858	0.775	0.949	0.003	0.006	AAA	High
LOC105376400	rs10795668	10	G-A	3	TNM12	766	2423	0.773	0.682	0.875	4.65E−05	1.38E−04	AAA	High
CCND1	rs9344	11	G-A	4	TNM12	343	740	1.366	1.134	1.646	0.001	0.002	AAA	High
ABCB1	rs1045642	7	C-T	5	Asian	1567	1857	0.866	0.786	0.955	0.004	0.007	ABA	Intermediate
CYP1A1	rs1048943	15	A-G	7	Caucasian	1779	1946	1.251	1.038	1.508	0.019	0.026	BAA	Intermediate
MDM2	rs2279744	12	T-G	4	Caucasian	1325	473	0.826	0.704	0.969	0.019	0.026	ABA	Intermediate
CCAT2	rs10090154	8	C-T	3	Caucasian	1084	1049	1.254	1.028	1.529	0.026	0.033	BAA	Intermediate
VDR	rs731236	12	T-C	3	Colon	677	578	1.291	1.06	1.573	0.011	0.017	BAA	Intermediate
PPARG	rs1801282	3	C-G	4	Colon	998	3368	0.772	0.619	0.963	0.022	0.029	BAA	Intermediate
TP53	rs1042522	17	G-C	5	TNM12	249	975	1.41	1.147	1.734	0.001	0.002	BAA	Intermediate
XRCC1	rs25487	19	G-A	3	TNM12	165	420	1.323	1.021	1.713	0.034	0.038	BBA	Intermediate
miR-196a2	rs11614913	12	C-T	3	TNM12	279	1249	0.823	0.682	0.994	0.043	0.045	ABA	Intermediate
MTHFR	rs1801133	1	C-T	4	TNM34	207	715	1.715	1.338	2.200	2.09E−05	6.39E−05	BAA	Intermediate
CCND1	rs9344	11	G-A	5	TNM34	340	860	1.254	1.046	1.503	0.014	0.021	ABA	Intermediate
CCAT2	rs6983267	8	G-T	8	Asian	11190	10699	0.846	0.791	0.906	1.43E−06	5.80E−06	ACA	Low
miR-27a	rs895819	19	T-C	3	Asian	1174	1212	1.237	1.096	1.395	0.001	0.002	AAC	Low
BMP4	rs4444235	14	T-C	4	Asian	4136	4765	1.089	1.026	1.156	0.005	0.009	ABC	Low
MMP9	rs3918242	20	C-T	4	Asian	702	888	0.754	0.605	0.939	0.012	0.018	BAC	Low
PTGS2	rs20417	1	G-C	3	Asian	1285	1740	1.422	1.08	1.872	0.012	0.018	BBC	Low
GREM1-SCG5	rs4779584	15	C-T	4	Asian	4122	4681	1.134	1.003	1.283	0.045	0.047	ACC	Low
C11orf92–C11orf93	rs3802842	11	A-C	11	Caucasian	12983	12139	1.139	1.095	1.184	6.32E−11	5.81E−10	ABC	Low
SMAD7	rs4464148	18	T-C	7	Caucasian	9020	7860	1.139	1.087	1.194	4.74E−08	3.11E−07	AAC	Low
CCAT2	rs6983267	8	G-T	17	Caucasian	21026	18580	0.85	0.798	0.904	2.97E−07	1.44E−06	ACA	Low
SMAD7	rs4939827	18	T-C	13	Caucasian	12657	11621	0.853	0.801	0.908	5.86E−07	2.69E−06	ACA	Low
CDH1	rs9929218	16	G-A	13	Caucasian	18291	19311	0.926	0.897	0.956	2.17E−06	7.67E−06	AAC	Low
RHPN2	rs10411210	19	C-T	12	Caucasian	19240	20177	0.859	0.799	0.925	5.25E−05	1.51E−04	ACA	Low
TERT	rs2736100	5	G-T	8	Caucasian	14486	15588	1.072	1.036	1.109	5.83E−05	1.62E−04	AAC	Low
NA	rs719725	9	A-C	12	Caucasian	10035	10281	0.935	0.898	0.973	0.001	0.002	AAC	Low
NQO1	rs1800566	16	C-T	9	Caucasian	4496	4794	1.119	1.039	1.206	0.003	0.006	AAC	Low
CDH1	rs16260	16	C-A	5	Caucasian	5603	6646	0.927	0.876	0.981	0.008	0.013	AAC	Low
TGF-β1	rs1800469	19	C-T	5	Caucasian	2910	3684	0.915	0.845	0.987	0.021	0.028	ABC	Low
MTR	rs1805087	1	A-G	10	Caucasian	6884	8873	0.936	0.884	0.991	0.023	0.029	ABC	Low
NA	rs11568820	12	G-A	4	Caucasian	2701	2721	1.107	1.011	1.213	0.028	0.035	AAC	Low
RFC/SLC19A1	rs1051266	21	A-G	5	Caucasian	1922	3142	0.915	0.843	0.992	0.032	0.037	AAC	Low
GREM1	rs16969681	15	C-T	5	Caucasian	6411	8146	1.175	1.003	1.378	0.046	0.047	ACA	Low
CCAT2	rs6983267	8	G-T	5	Colon	1593	2439	0.823	0.706	0.959	0.012	0.018	ACA	Low
CCND1	rs9344	11	G-A	8	Colon	877	2394	1.147	1.024	1.284	0.018	0.025	AAC	Low
MSH6	rs1042821	2	C-T	3	Colon	3448	5447	1.091	1.008	1.181	0.032	0.037	AAC	Low
SMAD7	rs4939827	18	T-C	4	colon	2259	2809	0.92	0.85	0.995	0.037	0.041	ABC	Low
ABCB1	rs1045642	7	C-T	5	Colon	469	635	1.198	1.009	1.423	0.039	0.042	AAC	Low
CCAT2	rs6983267	8	G-T	4	Rectum	652	1718	0.756	0.599	0.953	0.018	0.025	ACA	Low
CCAT2	rs6983267	8	G-T	4	TNM34	747	2185	0.834	0.739	0.941	0.003	0.006	AAC	Low

*Chr* chromosome, *FDR* false discovery rate

^a^Major alleles-minor alleles; Venice criteria: A (high), B (moderate), C (weak) credibility for three parameters (amount of evidence, heterogeneity and bias; see text and Additional file 1 for more details); level of evidence: overall level of summary evidence according to the Venice criteria

##### Results based-on ethnicity

**Fig. 2:**
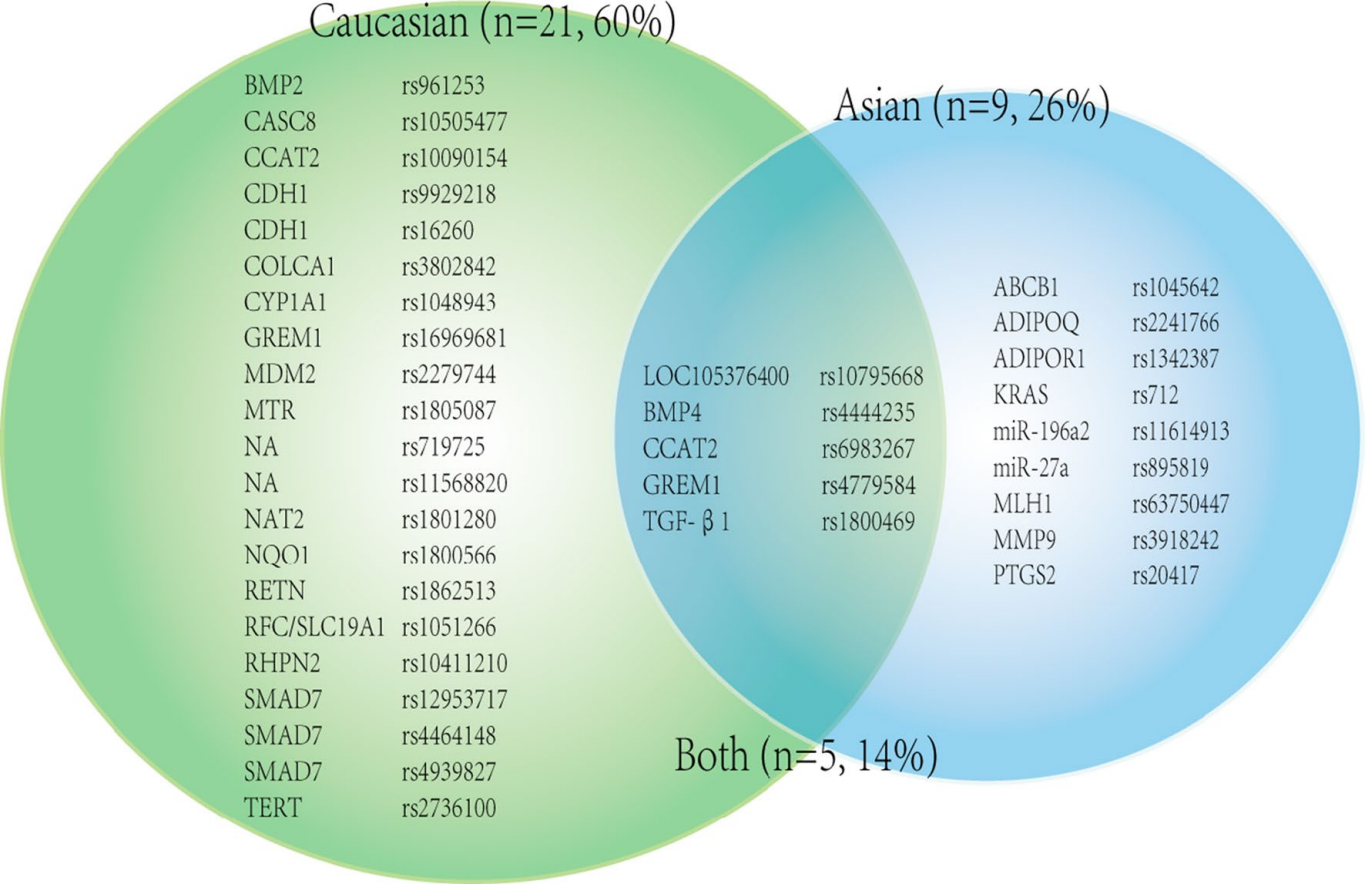
Venn diagram demonstrating the distribution of SNPs associated with colorectal cancer in subjects with Asian or Caucasian ethnicity or associated with both ancestries

Disparate race were mentioned in 35 significant associations (see Fig. 2). 21 (60%) of them were identified in Caucasian only (18 from Caucasian subgroup meta-analyses and 3 from primary meta-analyses which included only Caucasian ancestry), 9 (25.7%) of them were indicated in Asian only (8 from Asian subgroup meta-analyses and 1 from primary meta-analyses which only covered Asian ancestry), and 5 (14.3%) SNPs (all from subgroup meta-analyses) obtained their correlations in both Caucasian and Asian.

Totally 11 high quality SNPs were found in ethnicity subgroup analyses, 6 from Asian subgroup (TGF-β1 rs1800469, LOC105376400 rs10795668, KRAS rs712, ADIPOR1 rs1342387, ADIPOQ rs2241766 and miR-196a2 rs11614913) and 5 from Caucasian subgroup (BMP2 rs961253, BMP4 rs4444235, SMAD7 rs12953717, GREM1-SCG5 rs4779584 and LOC105376400 rs10795668).

#### Results based-on primary cancer site

**Fig. 3:**
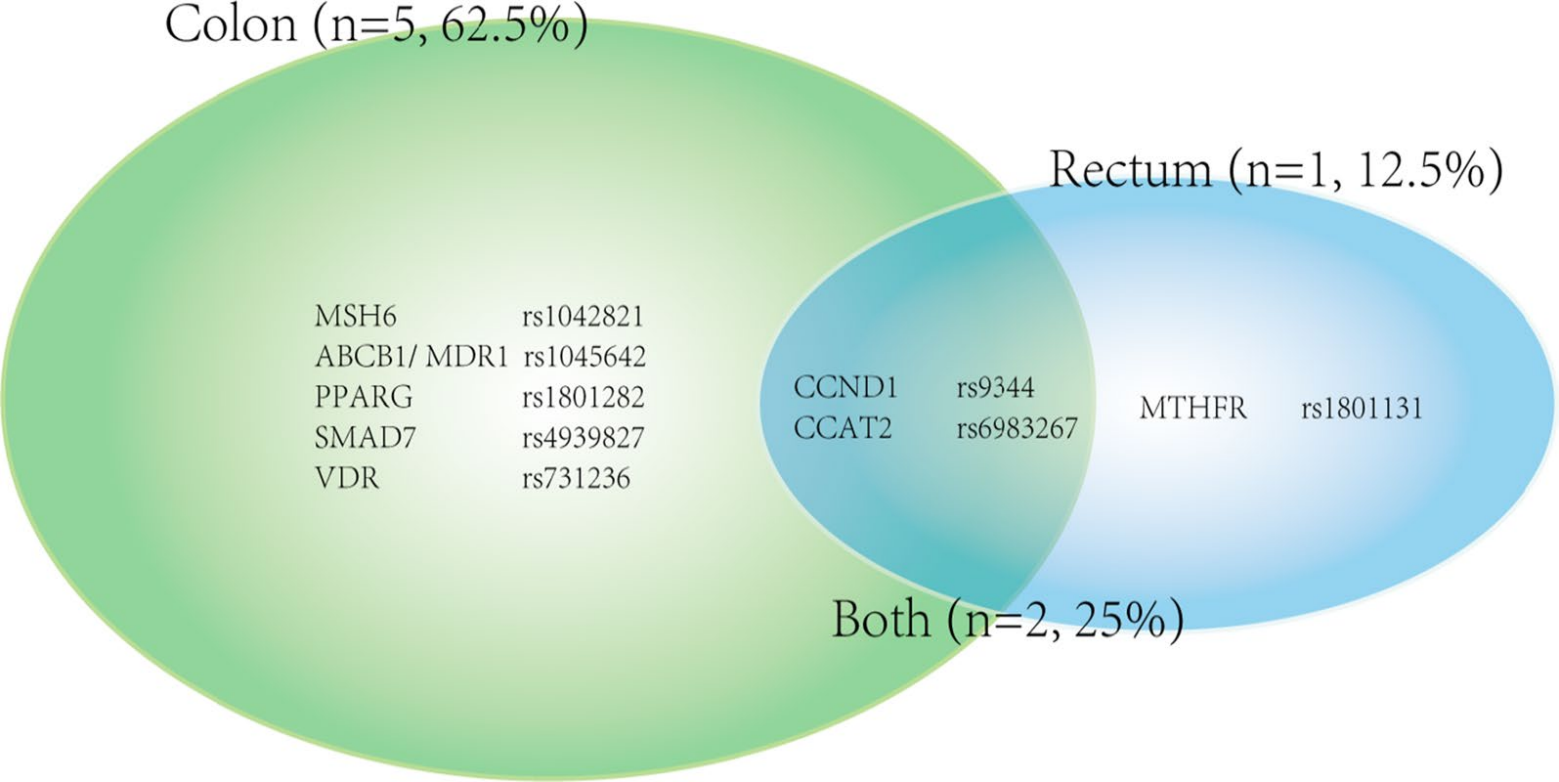
Venn diagram demonstrating the distribution of SNPs associated with colon or rectal cancer or associated with both primary cancer sites

Different cancer sites were mentioned in 8 significant SNPs (see Fig. 3). 5 (62.5%) of them showed their unique associations with colon cancer in subgroup analyses, 1 (12.5%) showed a sole association with rectum cancer in subgroup analysis, and 2 (25%) revealed their correlations with either colon or rectum cancer. Two high quality SNPs were found in rectum subgroup (CCND1 rs9344 and MTHFR rs1801131).

#### Results based-on cancer TNM stage

**Fig. 4:**
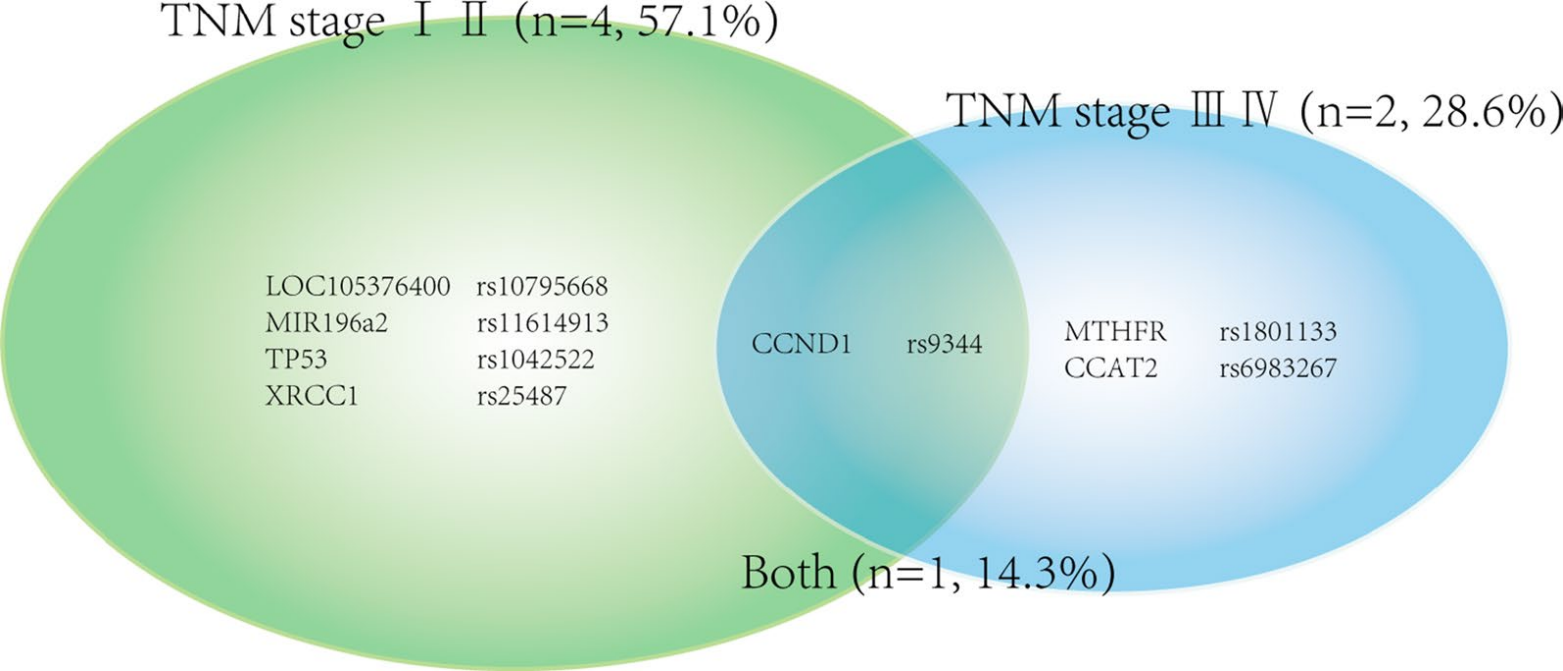
Venn diagram demonstrating the distribution of SNPs associated with specific coloretal cancer TNM stage (I II vs III IV) or associated with full stage

Subgroup meta-analyses of TNM stage demonstrated 7 SNPs with significant correlations (see Fig. 4). 4 (57.1%) of them simply correlated with TNM stage (I II), 2 (28.6%) of them related to TNM stage (III IV), and only 1 (14.3%) SNP showed it’s correlation with any TNM stage of CRC. Among the 7 significant SNPs, only 2 high quality SNPs (LOC105376400 rs10795668 and CCND1 rs9344) were identified.

#### Results based-on SNP location

**Fig. 5:**
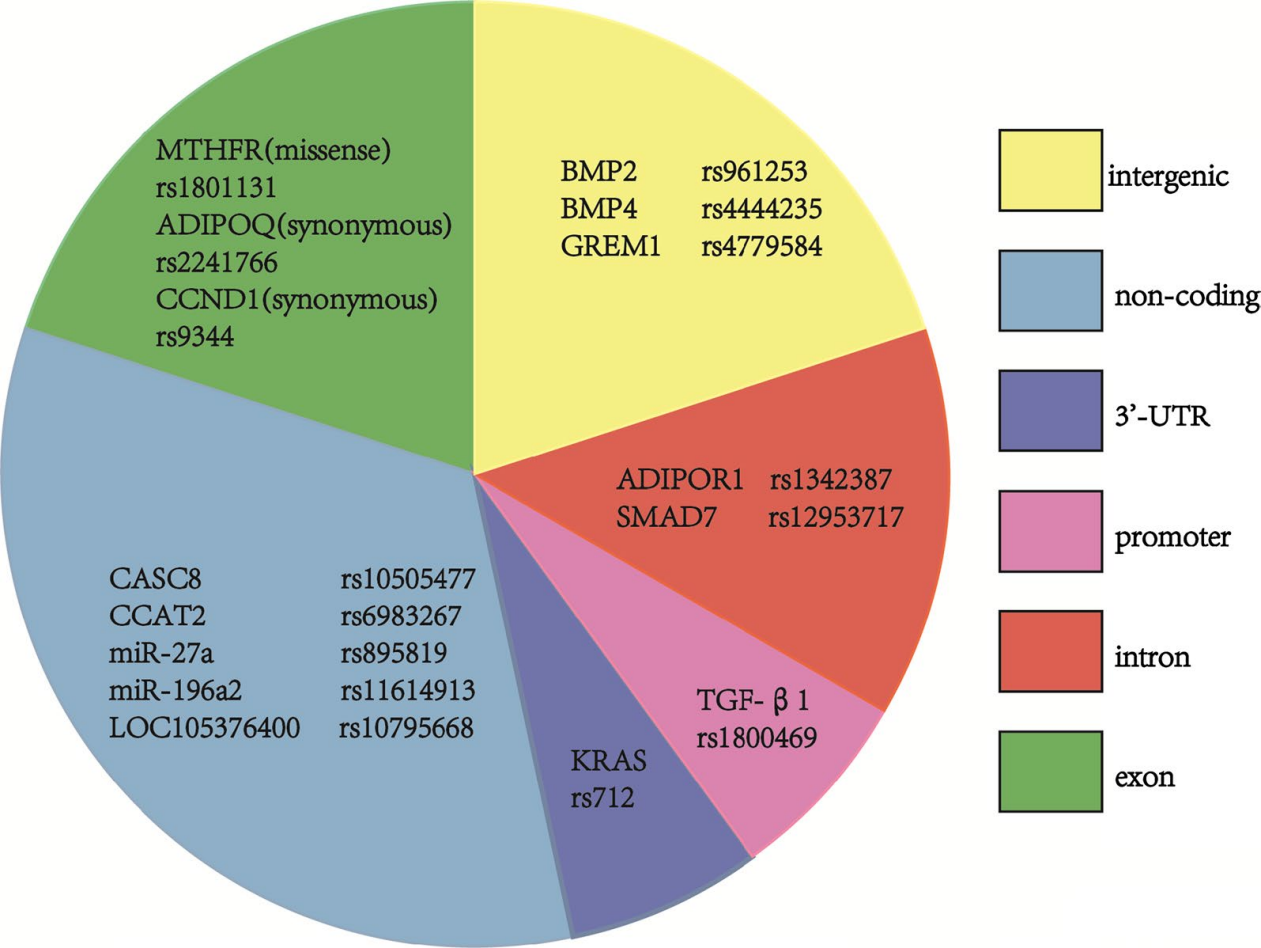
We artificially divided the 15 distinct high quality SNPs into six groups, based on their SNP loci on genes

From 25 high credibility significant associations, 15 distinct high quality SNPs were identified. In order to further explore the role of these high quality SNPs, we artificially divided them into different groups, based on their SNP loci (exon region, intron region, promoter region, downstream region, non-coding region and intergenic region), which was displayed in Fig. 5. What’s more, we also revealed the chromosome distribution of each high quality SNPs.

Of the 15 high quality SNPs, 3 are located in exon region (2 synonymous variants: ADIPOQ rs2241766 and CCND1 rs9344; one missense variant: MTHFR rs1801131), 2 in intron region (SMAD7 rs12953717 and ADIPOR1 rs1342387), one in promoter region (TGF-β rs1800469 SNP), one in 3′-UTR region (KRAS rs712) and 5 in non-coding region (CASC8 rs10505477, CCAT2 rs6983267, LOC105376400 rs10795668, miR-27a rs895819, miR-196a2 rs11614913). Distinct from those functional SNPs, there are still 3 high quality SNPs located in intergenic region (BMP2 rs961253, BMP4 rs4444235 and GREM1-SCG5 rs4779584).

The chromosomes distribution of each high quality SNPs were also displayed. In general, the SNPs are evenly and extensively distributed in half of the chromosomes including chromosome 1, 3, 8, 10, 11, 12, 14, 15, 18, 19, 20.

## Discussion

In this article, we systematically reviewed the associations between 160 SNPs in 96 distinct genes or chromosomal loci and predisposition to NHCRC or to subgroups identified by ethnicity (Asian vs Caucasian), primary cancer site (colon vs rectum), TNM stage (I II vs III IV) and SNP locations on genes, with the quality assessment of cumulative evidence, and 15 high quality SNPs were ultimately confirmed. Above all, innovations and strengths of the present study ought to be addressed. First, a most comprehensive evaluation of the literature in the field of genetic predisposition to NHCRC was conducted. Second, we first reported 20 SNPs in primary meta-analysis, 24 SNPs in “primary cancer site” subgroup analysis (15 for colon, 9 for rectum) and 10 SNPs in “TNM stage” subgroup analysis. Third, for exploring the functional roles of high quality SNPs on the NHCRC development, we first divided them into six different groups, based on SNP loci on genes. This study provides the latest evidence and clues for the genetic susceptibility to NHCRC. In spite of these strengths, limitations cannot be ignored. First, we only considered allelic genetic model because it was widely regarded as a conservative model between the dominant and recessive model [16]. Second, type I error might exist by utilizing same series in more than one meta-analysis. However, after calculating *q* values, the incidence of type I error could be minimized. Third, we didn’t analyze gene–gene or gene–environment interactions due to the insufficiency data. Future specialized studies should be designed to reveal their interactions.

### High quality SNPs with NHCRC risk

Facing the excessive SNPs with significant associations, it’s crucial to conduct a quality evaluation scientifically to those significant correlations. By utilizing the Venice criteria, we identified 15 high quality SNPs with 25 high credibility significant associations, which may act as promising genetic biomarkers for clinical NHCRC susceptibility screening.

For the whole population, 10 high quality SNPs were evaluated and shown in Table 1. Comparing our results with two published field meta-analyses [3, 4], we found that 8 of the 10 SNPs were assessed as high quality SNPs for the first time, which meant they were used to be non-high-quality SNPs (with intermediate or low credibility level evidence), or even unreported in the past. Interestingly, by observing the gene functions of these high quality SNPs, we noticed that half of them participated in TGF-β/Smad signaling pathway, including TGF-β, SMAD7, BMP2, BMP4 and GREM1. This discovery could indirectly verified the crucial role of TGF-β/Smad signaling pathway on CRC pathogenesis by regulating their target genes [17]. In addition, there were four other high quality SNPs in non-coding RNA (including 1 micro-RNA: miR-27a; 3 long non-coding RNA: CASC8, CCAT2 and LOC105376400), which revealed that the aberrant expression of non-coding RNA could also be tightly related to CRC diagnosis [18–20]. Moreover, there was also one high quality SNP in ADIPOQ (adiponectin) gene, reminding that the deficiency of adiponectin might be one of the fundamental risk factors for NHCRC [21, 22].

From the perspective of ethnicity, the apparent contrast between Caucasian and Asian population on the distribution of associated SNPs was presented in Fig. 2, which suggested that the molecular mechanism of CRC development couldn’t always be the same among different ethnicities. Of note, 6 high quality SNPs were evaluated in Asian subgroup, all of which were first identified as high quality SNPs for Asian population; while 5 high quality SNPs were evaluated in Caucasian subgroup, and 3 were newly identified for Caucasian population. Observing the gene functions of these SNPs, KRAS, an important oncogene, caught our attention. It participated in RAF/MEK/MAPK, ERK and AKT signal pathways, regulating the CRC cell proliferation and differentiation [23, 24].

From the aspect of primary cancer location, the different findings between colon and rectal cancer indicated that they not only differ in anatomic site, but also in molecular profile. A study illustrated that colon and rectal cancer differ in embryological origin, metastasis manner and mutational profile, requiring various neo-adjuvant treatment and surgical methods [25]. Nevertheless, none of the two publications have been concerned with the “primary cancer location” subgroup analyses. Herein, 2 high quality SNPs were demonstrated in rectal subgroup analysis. These results elucidated that the risk factors for rectum cancer development might be the aberrant expression of MTHFR (which leaded to abnormal folate metabolism [26–28]) or CCND1 (which could promote cell cycle G1/S transition [29, 30]).

From malignant level perspective, TNM stage subgroup was first analyzed in our study with a high positive rate (8/20, 40%) and the diversity between stage I II and III IV also exist. It illustrated that SNPs could not only predict the NHCRC development, but also remind the degree of malignancy, directing the physical test frequency for patient and the treatment for doctor. Based on the limited pathological parameters provided by researchers, only 20 SNPs were analyzed in this subgroup and 2 of them (LOC105376400 rs10795668 and CCND1 rs9344) were identified as high quality SNPs in TNM stage (I II). Further studies should pay more attention to the association between polymorphisms and NHCRC malignancy degree.

### Functional roles of high quality SNPs based on location

SNPs can influence the CRC susceptibility through complicated genetic and epigenetic mechanisms which depends on the their gene functions and their locations on genes. Hence, we arranged 15 high quality SNPs into different regions (including exon, intron, promoter, non-coding and also intergenic region) to focus on their feasible mechanisms on facilitating NHCRC development.

In exon region, the missense SNP (MTHFR rs1801131) make its contribution to the NHCRC by reducing the activity of enzyme [31, 32]. Besides, the prime mechanisms for synonymous SNPs are their influence on mRNA expression level by altering splicing or stability of mRNA (such as ADIPOQ rs2241766 and CCND1 rs9344) [33–36].

Indeed, SNPs in intron region probably exert larger effects on target genes than we hitherto thought, on account of the plenty of functional elements in this region, including cis-acting RNA elements, intron splice enhancers and intron splice silencers and so on [37]. However, high quality SNPs in this region are shown to be associated with mRNA expression level without precise interpretation (such as SMAD7 rs12953717 and rs4464148) [38]. Hence, the mechanisms of high quality intronic SNPs should not be ignored by further researchers and studies concerned with these SNPs are still found wanting.

Regarding the SNPs located in promoter region, it has revealed that they can alter the binding ability to transcription factors, affecting the transcriptional efficiency of genes (such as TGF-β1 rs1800469) [39]. Moreover, the 3′-UTR region of genes contain multiple microRNA binding sites. Hence, SNPs in this region are speculated to disrupt the microRNA binding sites, leading to an increased expression level of target genes (such as KRAS rs712, predicted by a bioinformatics website: ‘snpinfo.niehs.nih.gov’).

For SNPs in non-coding region, we found that high quality SNPs were detected in both microRNAs (miRNA) and long non-coding RNAs (lncRNA), which could indirectly participate in CRC cancerogenesis by interacting with encoding mRNA. SNPs in miRNA have a crucial influence on its synthesis and down-regulation (such as miR-196a2 rs11614913 and miR-27a rs895819) [20, 40, 41] and can also regulate the binding capacity to target genes (such as miR-196a2 rs11614913) [42]. In addition, SNPs in lncRNA can lead to an aberrant expression of lncRNA by disrupting its vital regulatory region (such as CASC8 rs1505477) [43], and regulate the expression level of target genes by modulating the binding of transcription factors (TFs) to its promoter region (such as CASC8 rs1505477, CCAT2 rs6983267 and LOC105376400 rs10795668) [44–47].

Furthermore, their were also three high quality SNPs: BMP2 rs961253, BMP4 rs4444235 and GREM1 rs4779584, not located in known genes. Further studies are required to explain their association with CRC risk. Additionally, data in our study revealed that high quality SNPs are diffused distributed in coding or non-coding region of chromosomes: 1, 3, 8, 10, 11, 12, 14, 15, 18, 19 and 20, which indicated the complicated molecular mechanisms for CRC generation involve numerous genomic and epigenomic variants.

## Conclusion and expectations

In this systematic review and large-scale meta-analysis, we identified 15 distinct high quality SNPs associated with NHCRC risk and first reported 20 SNPs in primary meta-analysis, 24 SNPs in subgroup analysis (15 for colon, 9 for rectum) and 10 SNPs in TNM stage subgroup analysis. The comprehensive survey in the field of genetic predisposition to sporadic colorectal cancer generalized the current situation of the study on NHCRC susceptibility SNPs, providing useful data for investigators to design future studies.

## Additional file

**Additional file 1: Table S1.** Detailed information of meta-analyses results for 25 high credibility significant associations. **Table S2.** Meta-analysis results: SNPs with non-significant associations to CRC risk.

## Abbreviations

CRC: colorectal cancer
HCRC: hereditary CRC
NHCRC: non-hereditary CRC
SNP: single nucleotide polymorphism
GWAS: genome-wide association studies
CGAS: candidate-gene association studies
VNTR: variable number of tandem repeat
ORs: odds ratios
CIs: confidence intervals
HWE: Hardy–Weinberg equilibrium
CIs: confidence intervals
FDR: false discovery rate
