# Supplementary material for: Single nucleotide polymorphisms and sporadic colorectal cancer susceptibility: a field synopsis and meta-analysis
Source: Cancer Cell Int. 2018 Oct 10;18:155. doi: 10.1186/s12935-018-0656-2 (PMC6180373; doi:10.1186/s12935-018-0656-2)
Supplement: Supplementary file 1 — Additional file 1: Table S1. Detailed information of meta-analyses results for 25 high credibility significant associations. Table S2. Meta-analysis results: SNPs with non-significant associations to CRC risk. [file 12935_2018_656_MOESM1_ESM.doc]

| Table S1 Detailed information of meta-analyses results for 25 high credibility significant associations | | | | | | | | | | | | | | | | | | | | | | |
| --- | --- | --- | --- | --- | --- | --- | --- | --- | --- | --- | --- | --- | --- | --- | --- | --- | --- | --- | --- | --- | --- | --- |
| Gene | SNPs | Risk allele | Subgroup-race | datasets | OR | CI-U | CI-L | P | FDR | Venice criteria grade | Cumulative evidence of association | Amount of Evidence | Amount of evidence grade | Consistency of replication | | Protection from bias | | Initial study influence | | 0.87<OR<1.15 | Publication bias | |
|  |  |  |  |  |  |  |  |  |  |  |  |  |  | I^2^ (%) | Replication grade | Reason for bias | Bias grade | OR (95% CI) | *P* value |  | Egger *P* | Begg *P* |
| BMP2 | rs961253 | C-A | All | 23 | 1.121 | 1.098 | 1.144 | 7.771E-27 | 7.149E-25 | AAA | High | 56880 | A | 20.4 | A | OR, Egger, Begg | Aǁ | 1.120(1.092-1.149) | 5.848E-18 | Yes | 0.029 | 0.016 |
| CASC8 | rs10505477 | T-C | All | 13 | 0.856 | 0.828 | 0.886 | 9.272E-19 | 2.843E-17 | AAA | High | 25103 | A | 7.4 | A | NA | A | 0.848（0.817-0.880） | 3.552E-18 | No | 0.515 | 0.393 |
| BMP4 | rs4444235 | T-C | All | 27 | 1.083 | 1.061 | 1.105 | 8.238E-15 | 1.895E-13 | AAA | High | 74854 | A | 14.9 | A | OR | Aǁ | 1.071(1.046-1.096) | 9.891E-09 | Yes | 0.104 | 0.104 |
| SMAD7 | rs12953717 | C-T | All | 9 | 1.163 | 1.118 | 1.209 | 4.93E-14 | 7.996E-13 | AAA | High | 18422 | A | 7.9 | A | NA | A | 1.150(1.069-1.237) | 1.831E-04 | No | 0.904 | 0.532 |
| CCAT2 | rs6983267 | G-T | All | 26 | 0.845 | 0.807 | 0.884 | 3.354E-13 | 3.857E-12 | AAA | High | 63737 | A | 66.9 | Aǁ | NA | A | 0.851(0.805-0.898) | 6.637E-09 | No | 0.396 | 0.545 |
| TGF-β1 | rs1800469 | C-T | All | 21 | 0.911 | 0.887 | 0.936 | 1.588E-11 | 1.623E-10 | AAA | High | 52072 | A | 22 | A | OR | Aǁ | 0.910(0.885-0.935) | 8.641E-12 | Yes | 0.954 | 0.365 |
| LOC105376400 | rs10795668 | G-A | All | 8 | 0.837 | 0.791 | 0.886 | 8.433E-10 | 5.270E-09 | AAA | High | 7390 | A | 2.5 | A | NA | A | 0.856(0.799-0.916) | 7.990E-06 | No | 0.272 | 0.458 |
| GREM1-SCG5 | rs4779584 | C-T | All | 16 | 1.154 | 1.093 | 1.217 | 1.687E-07 | 8.622E-07 | AAA | High | 31236 | A | 56.5 | Aǁ | NA | A | 1.133（1.091-1.176） | 5.342E-11 | No | 0.364 | 0.857 |
| ADIPOQ | rs2241766 | T-G | All | 7 | 1.216 | 1.115 | 1.326 | 9.330E-06 | 3.066E-05 | AAA | High | 2986 | A | 0 | A | NA | A | 1.202(1.101-1.313) | 4.058E-05 | No | 0.733 | 0.764 |
| miR-27a | rs895819 | T-C | All | 5 | 1.192 | 1.076 | 1.32 | 0.001 | 2.091E-03 | AAA | High | 2263 | A | 9.9 | A | NA | A | 1.225(1.099-1.367) | 2.603E-04 | No | 0.967 | 1 |
| TGF-β1 | rs1800469 | C-T | Asian | 16 | 0.911 | 0.885 | 0.938 | 2.432E-10 | 2.034E-09 | AAA | High | 47965 | A | 17.2 | A | OR | Aǁ | NA | NA | Yes | 0.83 | 0.653 |
| LOC105376400 | rs10795668 | G-A | Asian | 4 | 0.803 | 0.741 | 0.872 | 1.369E-07 | 7.409E-07 | AAA | High | 3620 | A | 4.6 | A | NA | A | 0.817(0.715-0.934) | 0.003 | No | 0.533 | 0.174 |
| KRAS | rs712 | G-T | Asian | 3 | 1.405 | 1.226 | 1.61 | 9.986E-07 | 4.375E-06 | AAA | High | 1079 | A | 0 | A | NA | A | NA | NA | No | 0.296 | 0.602 |
| ADIPOR1 | rs1342387 | G-A | Asian | 3 | 0.779 | 0.697 | 0.87 | 0.000009218 | 3.066E-05 | AAA | High | 2118 | A | 19.8 | A | NA | A | NA | NA | No | 0.793 | 0.602 |
| ADIPOQ | rs2241766 | T-G | Asian | 5 | 1.215 | 1.111 | 1.328 | 6.030E-05 | 1.632E-04 | AAA | High | 2849 | A | 0 | A | NA | A | NA | NA | No | 0.79 | 0.463 |
| miR-196a2 | rs11614913 | C-T | Asian | 4 | 0.836 | 0.757 | 0.922 | 3.537E-04 | 8.794E-04 | AAA | High | 3590 | A | 21.6 | A | NA | A | 0.859(0.769-0.960) | 0.008 | No | 0.321 | 0.497 |
| BMP2 | rs961253 | C-A | Caucasian | 21 | 1.115 | 1.092 | 1.139 | 3.569E-24 | 1.642E-22 | AAA | High | 54772 | A | 0 | A | OR | Aǁ | 1.112(1.083-1.141) | 2.536E-15 | Yes | 0.195 | 0.103 |
| BMP4 | rs4444235 | T-C | Caucasian | 22 | 1.086 | 1.063 | 1.11 | 5.215E-14 | 7.996E-13 | AAA | High | 64676 | A | 0 | A | OR, Egger, Begg | Aǁ | 1.073(1.046-1.102) | 8.237E-08 | Yes | 0.03 | 0.042 |
| SMAD7 | rs12953717 | C-T | Caucasian | 8 | 1.161 | 1.116 | 1.208 | 1.163E-13 | 1.529E-12 | AAA | High | 18242 | A | 16.2 | A | NA | A | 1.144(1.061-1.233) | 4.299E-04 | No | 0.904 | 0.532 |
| GREM1-SCG5 | rs4779584 | C-T | Caucasian | 12 | 1.16 | 1.092 | 1.232 | 0.000001452 | 5.804E-06 | AAA | High | 16986 | A | 57.9 | Aǁ | NA | A | 1.141（1.093-1.190） | 1.42E-09 | No | 0.254 | 0.891 |
| LOC105376400 | rs10795668 | G-A | Caucasian | 4 | 0.87 | 0.804 | 0.942 | 0.001 | 2.091E-03 | AAA | High | 3770 | A | 0 | A | NA | A | NA | NA | Yes | 0.375 | 0.174 |
| CCND1 | rs9344 (A870G) | G-A | rectum | 7 | 1.272 | 1.126 | 1.436 | 1.053E-04 | 2.767E-04 | AAA | High | 3113 | A | 12.1 | A | NA | A | 1.267(1.120-1.434) | 1.797E-04 | No | 0.425 | 0.652 |
| MTHFR | rs1801131 | A-C | rectum | 6 | 0.858 | 0.775 | 0.949 | 0.003 | 5.520E-03 | AAA | High | 2346 | A | 16.6 | A | NA | A | 0.855(0.771-0.948) | 0.003 | No | 0.61 | 0.573 |
| LOC105376400 | rs10795668 | G-A | TNM12 | 3 | 0.773 | 0.682 | 0.875 | 4.654E-05 | 1.381E-04 | AAA | High | 2280 | A | 0 | A | NA | A | NA | NA | No | 0.127 | 0.117 |
| CCND1 | rs9344 | G-A | TNM12 | 4 | 1.366 | 1.134 | 1.646 | 0.001 | 2.091E-03 | AAA | High | 1152 | A | 0 | A | NA | A | 1.367(1.113-1.679) | 0.003 | No | 0.19 | 0.174 |
| Note: Aǁ, Venice criteria does not suitable for meta-analysis results (*P*<1×10 ^-7^ ) after the removal of the initial study | | | | | | | | | | | | | | | | | | | | | | |

| Table S2 Meta-analysis results: SNPs with non-significant associations to CRC risk | | | | | | | | | | |
| --- | --- | --- | --- | --- | --- | --- | --- | --- | --- | --- |
| Gene | SNPs | Alleles^#^ | Datasets | Cases | Controls | Subgroups | OR | CI-U | CI-L | P |
| miR-499 | rs3746444 | T-C | 3 | 814 | 1174 | All | 1.070 | 0.907 | 1.262 | 0.425 |
| miR-149 | rs2292832 | C-T | 4 | 1492 | 1550 | All | 0.945 | 0.848 | 1.052 | 0.299 |
| CTLA4 | rs231775 | A-G | 5 | 537 | 1126 | All | 1.070 | 0.910 | 1.259 | 0.411 |
| PTGS2 | rs20417 | G-C | 12 | 6360 | 8346 | All | 1.069 | 1.000 | 1.143 | 0.051 |
| PTGS2 | rs5275 | C-T | 8 | 5532 | 7549 | All | 0.997 | 0.947 | 1.050 | 0.915 |
| PTGS2 | rs2745557 | G-A | 4 | 4000 | 5525 | All | 1.009 | 0.935 | 1.089 | 0.810 |
| PTGS2 | rs5277 | G-C | 6 | 2583 | 3212 | All | 1.025 | 0.927 | 1.134 | 0.625 |
| CRP | rs1800947 | G-C | 3 | 2853 | 3381 | All | 1.013 | 0.871 | 1.177 | 0.864 |
| IL-4 | rs2243250 | C-T | 3 | 680 | 1025 | All | 0.972 | 0.811 | 1.163 | 0.757 |
| ADIPOQ | rs266729 | C-G | 4 | 1851 | 2654 | All | 0.976 | 0.889 | 1.072 | 0.616 |
| ADIPOQ | rs1501299 | C-A | 8 | 2081 | 3287 | All | 0.967 | 0.887 | 1.053 | 0.439 |
| ADIPOQ | rs822395 | A-C | 4 | 1438 | 1950 | All | 0.964 | 0.854 | 1.088 | 0.551 |
| XPC | rs2228001 | A-C | 12 | 4567 | 35581 | All | 1.037 | 0.956 | 1.125 | 0.384 |
| IRS2 | rs1805097 | G-A | 4 | 4072 | 4994 | All | 0.954 | 0.897 | 1.015 | 0.134 |
| MGMT | rs2308321 | A-G | 3 | 832 | 3507 | All | 1.160 | 0.966 | 1.396 | 0.111 |
| CYP1A2 | rs2069514 | G-A | 4 | 1223 | 1232 | All | 0.993 | 0.860 | 1.146 | 0.920 |
| CYP1B1 | rs1056836 | G-C | 8 | 7267 | 8003 | All | 0.984 | 0.940 | 1.030 | 0.487 |
| CYP1B1 | rs1800440 | A-G | 7 | 6798 | 7429 | All | 0.971 | 0.914 | 1.032 | 0.348 |
| XPD | rs13181 | A-C | 17 | 5823 | 8798 | All | 1.028 | 0.974 | 1.085 | 0.311 |
| SOD2 | rs4880 | C-T | 3 | 1677 | 1563 | All | 0.966 | 0.876 | 1.065 | 0.484 |
| IGFBP3 | rs2854744 | C-A | 9 | 6469 | 9405 | All | 1.017 | 0.970 | 1.067 | 0.476 |
| VEGF | rs2010963 | G-C | 5 | 1890 | 1762 | All | 0.969 | 0.880 | 1.067 | 0.520 |
| SHMT1 | rs1979277 | C-T | 5 | 2323 | 3374 | All | 0.997 | 0.919 | 1.081 | 0.942 |
| MTR | rs1805087 | A-G | 21 | 11536 | 15930 | All | 0.970 | 0.928 | 1.013 | 0.162 |
| IL-10 | rs1800896 | A-G | 6 | 1299 | 2035 | All | 0.977 | 0.875 | 1.091 | 0.682 |
| EPHX1 | rs1051740 | T-C | 14 | 7797 | 9647 | All | 1.007 | 0.962 | 1.055 | 0.760 |
| CYP2C9 | rs1799853 | C-T | 5 | 3853 | 4561 | All | 0.944 | 0.864 | 1.031 | 0.203 |
| IL-8 | rs4073 | T-A | 5 | 1242 | 2051 | All | 0.966 | 0.873 | 1.069 | 0.502 |
| NAT2 | rs1799931 | G-A | 3 | 2348 | 2847 | All | 1.130 | 0.878 | 1.453 | 0.343 |
| NAT2 | rs1143627 | T-C | 3 | 827 | 1235 | All | 1.000 | 0.876 | 1.142 | 0.997 |
| TLR-4 | rs4986790 | A-G | 7 | 1190 | 1506 | All | 1.166 | 0.933 | 1.458 | 0.178 |
| LEPR | rs1137101 | A-G | 3 | 912 | 979 | All | 1.024 | 0.901 | 1.165 | 0.715 |
| MTHFD1 | rs2236225 | C-T | 8 | 5528 | 8645 | All | 0.984 | 0.937 | 1.033 | 0.510 |
| RFC/SLC19A1 | rs1051266 | A-G | 8 | 4915 | 7590 | All | 0.995 | 0.945 | 1.048 | 0.859 |
| NBS1 | rs1805794 | G-C | 4 | 1560 | 1675 | All | 0.999 | 0.903 | 1.104 | 0.979 |
| XPF | rs1800067 | G-A | 4 | 1023 | 1280 | All | 1.026 | 0.821 | 1.281 | 0.822 |
| MTHFD1 | rs1950902 | G-A | 4 | 1859 | 2938 | All | 0.902 | 0.809 | 1.005 | 0.062 |
| VDR/CDX-2 | rs2238136 | G-A | 3 | 1291 | 1228 | All | 1.056 | 0.937 | 1.190 | 0.372 |
| UGT1A6 | rs1105879 | T-G | 3 | 2843 | 3802 | All | 1.010 | 0.941 | 1.084 | 0.784 |
| AXIN2 | rs2240308 | G-A | 4 | 1254 | 1271 | All | 1.116 | 0.997 | 1.249 | 0.056 |
| PEDF | rs10808556 | T-C | 4 | 2436 | 3032 | All | 1.124 | 1.040 | 1.216 | 0.003 |
| PEDF | rs7013278 | C-T | 3 | 1063 | 1040 | All | 1.064 | 0.940 | 1.204 | 0.326 |
| PEDF | rs7000448 | C-T | 3 | 1064 | 1040 | All | 0.986 | 0.870 | 0.118 | 0.829 |
| CCAT2 | rs1447295 | C-A | 3 | 1072 | 1045 | All | 1.178 | 0.972 | 1.429 | 0.096 |
| CCAT2 | rs7857826 | T-A | 3 | 1072 | 1047 | All | 0.973 | 0.861 | 1.099 | 0.658 |
| COX-2 | rs5275 | C-T | 7 | 5111 | 7069 | Caucasian | 1.002 | 0.949 | 1.057 | 0.943 |
| COX-2 | rs2745557 | G-A | 3 | 3580 | 5044 | Caucasian | 1.020 | 0.941 | 1.105 | 0.632 |
| APC | rs459552 | A-T | 10 | 8833 | 9532 | Caucasian | 0.958 | 0.912 | 1.006 | 0.087 |
| IL-10 | rs1800896 | A-G | 5 | 924 | 1653 | Caucasian | 0.950 | 0.847 | 1.065 | 0.375 |
| EPHX1 | rs1051740 | T-C | 10 | 5186 | 6288 | Caucasian | 1.006 | 0.949 | 1.065 | 0.852 |
| MTHFD1 | rs2236225 | C-T | 5 | 3031 | 4766 | Caucasian | 1.007 | 0.943 | 1.074 | 0.844 |
| ADH1B/ADH2 | rs1229984 | A-G | 3 | 2439 | 3223 | Caucasian | 0.929 | 0.824 | 1.047 | 0.227 |
| COX-2 | rs20417 | G-C | 9 | 5075 | 6606 | Caucasian | 1.050 | 0.980 | 1.125 | 0.165 |
| hOGG1 | rs1052133 | C-G | 3 | 4343 | 3810 | Caucasian | 1.026 | 0.951 | 1.106 | 0.509 |
| IGFBP3 | rs2854744 | C-A | 4 | 2069 | 3939 | Caucasian | 1.028 | 0.947 | 1.117 | 0.507 |
| VDR | rs1544410 | G-A | 9 | 6836 | 6825 | Caucasian | 0.953 | 0.908 | 1.000 | 0.052 |
| XPC | rs2228001 | A-C | 7 | 2188 | 32649 | Caucasian | 1.032 | 0.958 | 1.113 | 0.405 |
| XPD | rs1799793 | G-A | 4 | 2130 | 2686 | Caucasian | 1.002 | 0.920 | 1.091 | 0.961 |
| XPG | rs17655 | G-C | 4 | 1041 | 1411 | Caucasian | 1.083 | 0.939 | 1.249 | 0.272 |
| MIR196a2 | rs11614913 | C-T | 4 | 707 | 1116 | Caucasian | 1.130 | 0.980 | 1.302 | 0.092 |
| MTRR | rs1801394 | A-G | 11 | 5349 | 7904 | Caucasian | 1.044 | 0.993 | 1.098 | 0.089 |
| CTLA4 | rs231775 | A-G | 4 | 413 | 719 | Caucasian | 0.993 | 0.820 | 1.201 | 0.940 |
| XRCC1 | rs25487 | G-A | 15 | 2522 | 4702 | Caucasian | 1.045 | 0.966 | 1.130 | 0.273 |
| COX-2 | rs5277 | G-C | 5 | 2162 | 2732 | Caucasian | 1.030 | 0.924 | 1.149 | 0.589 |
| ADIPOQ | rs1501299 | C-A | 4 | 1087 | 1654 | Caucasian | 1.022 | 0.908 | 1.151 | 0.714 |
| PPARG | rs1801282 | C-G | 11 | 8290 | 10647 | Caucasian | 0.957 | 0.897 | 1.020 | 0.177 |
| VEGF | rs2010963 | G-C | 3 | 1035 | 857 | Caucasian | 1.049 | 0.912 | 1.205 | 0.504 |
| SHMT1 | rs1979277 | C-T | 3 | 645 | 1287 | Caucasian | 0.972 | 0.840 | 1.125 | 0.706 |
| IL-10 | rs1800872 | C-A | 6 | 1133 | 1976 | Caucasian | 1.032 | 0.912 | 1.168 | 0.622 |
| EPHX1 | rs2234922 | A-G | 10 | 3263 | 3936 | Caucasian | 1.013 | 0.930 | 1.103 | 0.772 |
| CYP2C9 | rs1799853 | C-T | 5 | 3853 | 4561 | Caucasian | 0.944 | 0.864 | 1.031 | 0.203 |
| CYP2C9 | rs1057910 | A-C | 3 | 2363 | 2865 | Caucasian | 0.943 | 0.811 | 1.097 | 0.447 |
| TP53 | rs1042522 | G-C | 16 | 3186 | 4664 | Caucasian | 1.005 | 0.934 | 1.081 | 0.900 |
| TLR-4 | rs4986790 | A-G | 6 | 1000 | 1266 | Caucasian | 1.137 | 0.898 | 1.440 | 0.287 |
| NBS1 | rs1805794 | G-C | 3 | 797 | 783 | Caucasian | 0.920 | 0.794 | 1.066 | 0.268 |
| hOGG1 | rs1052133 | C-G | 7 | 2093 | 4068 | Asian | 1.009 | 0.993 | 1.101 | 0.819 |
| RHPN2 | rs10411210 | C-T | 3 | 3059 | 3103 | Asian | 0.926 | 0.846 | 1.013 | 0.092 |
| CDH1 | rs9929218 | G-A | 4 | 4168 | 4768 | Asian | 0.957 | 0.886 | 1.033 | 0.257 |
| XRCC3 | rs861539 | C-T | 3 | 1384 | 1341 | Asian | 0.887 | 0.757 | 1.038 | 0.134 |
| MIR-149 | rs2292832 | C-T | 3 | 1332 | 1372 | Asian | 0.948 | 0.846 | 1.063 | 0.360 |
| MTRR | rs1801394 | A-G | 4 | 1212 | 1617 | Asian | 1.008 | 0.897 | 1.133 | 0.891 |
| XRCC1 | rs25489 | G-A | 3 | 1213 | 1337 | Asian | 1.069 | 0.896 | 1.274 | 0.459 |
| ADIPOQ | rs822395 | A-C | 3 | 1245 | 1754 | Asian | 0.943 | 0.826 | 1.077 | 0.388 |
| CYP1A2 | rs2069514 | G-A | 3 | 895 | 937 | Asian | 0.993 | 0.859 | 1.149 | 0.925 |
| MTHFR | rs1801131 | A-C | 12 | 3026 | 4200 | Asian | 0.922 | 0.848 | 1.003 | 0.059 |
| XPD | rs13181 | A-C | 4 | 1535 | 2425 | Asian | 1.117 | 0.962 | 1.297 | 0.148 |
| EGF | rs4444903 | G-A | 3 | 572 | 607 | Asian | 0.844 | 0.706 | 1.008 | 0.061 |
| MTR | rs1805087 | A-G | 5 | 1465 | 2236 | Asian | 1.035 | 0.916 | 1.169 | 0.582 |
| TNF-α | rs1800629 | G-A | 6 | 1784 | 3467 | Asian | 0.991 | 0.850 | 1.155 | 0.904 |
| ERCC1 | rs11615 | C-T | 3 | 696 | 760 | Asian | 1.125 | 0.964 | 1.313 | 0.134 |
| MTHFR | rs1801131 | A-C | 3 | 2413 | 3021 | Mixed | 0.939 | 0.865 | 1.018 | 0.128 |
| MTR | rs1805087 | A-G | 5 | 2948 | 4499 | Mixed | 1.000 | 0.920 | 1.087 | 0.998 |
| MTRR | rs1801394 | A-G | 3 | 1609 | 2050 | Mixed | 1.063 | 0.969 | 1.167 | 0.198 |
| IGFBP3 | rs2854744 | C-A | 3 | 2886 | 3934 | colon | 0.994 | 0.926 | 1.067 | 0.863 |
| hOGG1 | [rs1052133](https://www.ncbi.nlm.nih.gov/projects/SNP/snp_ref.cgi?rs=1052133) | C-G | 3 | 1966 | 2683 | colon | 1.054 | 0.955 | 1.164 | 0.295 |
| XPD | rs13181 | A-C | 3 | 1976 | 2776 | colon | 0.972 | 0.892 | 1.059 | 0.515 |
| MTR | rs1805087 | A-G | 4 | 3260 | 3812 | colon | 0.956 | 0.879 | 1.039 | 0.287 |
| XPC | rs2228001 | A-C | 3 | 731 | 30610 | colon | 1.105 | 0.981 | 1.245 | 0.100 |
| NQO1 | rs1800566 | C-T | 3 | 408 | 1149 | colon | 1.043 | 0.846 | 1.287 | 0.692 |
| TNF | rs1800629 | G-A | 5 | 996 | 2473 | colon | 0.922 | 0.777 | 1.094 | 0.353 |
| VDR | rs1544410 | G-A | 3 | 1837 | 2747 | rectum | 0.934 | 0.852 | 1.025 | 0.152 |
| XPC | rs2228001 | A-C | 3 | 465 | 30610 | rectum | 1.115 | 0.962 | 1.293 | 0.148 |
| CRP | rs1205 | C-T | 3 | 1074 | 1362 | rectum | 1.071 | 0.951 | 1.207 | 0.260 |
| TNF | rs1800629 | G-A | 4 | 618 | 2293 | rectum | 0.886 | 0.718 | 1.094 | 0.260 |
| VDR | rs2228570 | C-T | 6 | 2131 | 4203 | rectum | 1.008 | 0.931 | 1.091 | 0.849 |
| CCAT2 | rs6983267 | G-T | 4 | 1170 | 2185 | TNM12 | 0.923 | 0.834 | 1.021 | 0.121 |
| MTHFR | rs1801133 | C-T | 4 | 155 | 715 | TNM12 | 0.991 | 0.745 | 1.317 | 0.950 |
| CDHN2A | rs10795668 | G-A | 3 | 648 | 2423 | TNM34 | 0.883 | 0.776 | 1.006 | 0.061 |
| XRCC1 | rs25487 | G-A | 3 | 166 | 420 | TNM34 | 0.939 | 0.720 | 1.224 | 0.639 |
| CTLA4 | rs231775 | A-G | 3 | 116 | 698 | TNM34 | 1.229 | 0.907 | 1.665 | 0.184 |
| Note, ^#^, Major alleles-minor alleles | | | | | | | | | | |
